# Supplementary material for: Multilevel analysis of continuation of maternal healthcare services utilization and its associated factors in Ethiopia: A cross-sectional study
Source: PLOS Glob Public Health. 2022 May 24;2(5):e0000517. doi: 10.1371/journal.pgph.0000517 (PMC10022002; doi:10.1371/journal.pgph.0000517)
Supplement: Attachment — Submitted filename: Response to Reviewers .docx [file pgph.0000517.s001.docx]

**Point-by-point response to the reviewers’ comments**

**Title:** Multilevel Analysis of Continuation of Maternal Healthcare Services Utilization and Its Associated Factors in Ethiopia: A Cross-Sectional Study (PGPH-D-21-00920)

**Journal Name:** PLOS Global Public Health

Dear Editor of PLOS Global Public Health Journal,

I have revised my manuscript as per the constructive comments of editor and reviewers. I did not carry out any additional analysis for this manuscript. I have checked that the manuscript conforms to the journal submission guidelines. I would like to appreciate all reviewers for providing very constructive and detail comments on my manuscript. I have responded to all comments and questions raised by each editor/reviewer point by point as following:

**Editor comment**

**Journal Requirements**:

Please update the completed 'Competing Interests' statement, including any COIs declared by your co-authors. If you have no competing interests to declare, please state "The authors have declared that no competing interests exist".

**Response**: Thank you, I have done it in the manuscript according to your comment.

**Reviewers' comments**

**Reviewer #1**

**Comment 1:** In the abstract: Check the use of acronyms please check conclusions in the abstracts as it is difficult to understand "targeting residence”,as it is not clear from the results.

**Response 1**: concerning acronyms: I have tried to narrate acronyms in the abstract at their first entry and I have used the acronyms for the subsequent use because of limited word counts in the abstract. I have edited comment on the conclusion part of abstract and highlighted in the manuscript.

**Comment 2**: In the **introduction** please clarify that in Ethiopia until the best of the knowledge CoC has not been studied

**Response 2:** Based on my database search, I found many studies conducted on ANC, or skilled delivery or postnatal care separately without considering their continuation on the continuum of care. Therefore, this is one of my justification for this study in addition to applying multilevel modeling.

**Comment 3**: Material and Methods

3.1. Please state if the survey was conducted during 2016 and addresses women with live births five years earlier this means 2011-2016? Please define clearly the period. Also, it is not clear wather you used informetion that already existed in the survey or tracked eligible participants and obtained the information after 2016. A more in detail description of the survey and the information collected would be great to better understand what was done and how

**Response 3.1**: It is known that DHS conducted every five years that means it collects data of the last five years from the year of current survey (2016-2011). So you are correct. I have used data already exist in the survey.

**Response 3.2**: Yes, as you said it is better if the detail description of the survey stated here in the manuscript. However, the limited word counts to write manuscript and DHS is a common and know survey for everybody. I gave some description about survey and prefer to cite reference to check the detail description of the survey for the readers. So the reference added to the manuscript

**Comment 4:** Results: What is higher birth order? Number of children? please clarify what you are referring!

**Response4:** refers to **the order a child is born in their family** first-born, second-born… etc. In this study higher birth order indicates those women with more than 3^rd^ birth order. Birth order is not always equal to number of children that indicates alive children at the time of interview. I have edited “higher birth order” in the manuscript for purpose of clarity.

**Comment 5:** Discussion: Better start with the study results

**Response 5:** Thank you, some of the reviewers recommend to start discussion with study objective and study result. I wrote the discussion based on that recommendation and add some finding according to your comment then highlighted in the manuscript.

**Comment 6**: Check english for example: line 232 in page 11 ANC+ visits are higher (include the verb please!) in line 232 and 233 instead of pregnancy wanted should be planned pregnancies right ?

**Response 6:** Thank you, I have corrected issue related to the mentioned line and issue related to comparison group**.**

**Comment 7:** What are the strengths of the study? Only limitations?

**Response 7:** I have added the strength of the study to the manuscript

**Reviewer #2**

**Introduction**

**Comment 1:** - The authors need to address why specifically this study is needed, as there remains a plethora of studies, particularly in the factors associated with the individual component of continuation of maternal health service utilization.

**Response 1:** Thank you dear reviewer! Continuation of maternal healthcare service from pregnancy to postnatal is crucial to improve maternal, infant health as well as to reduce morbidity and mortality. However, ample number of mothers did not continue up to postnatal service even those come to health facility for maternal health service and received at least first ANC. The main research question is why at least those mothers joined health facility will not continue to receive maternal healthcare service at each stage (pregnancy stage, delivery stage and postnatal stage? I have designed three outcome variables: Received ANC4+; received both ANC4+ and SBA; received all ANC4+, and SBA and PNC. So this study aimed to determine magnitude of continuation at each stage/level and its associated factors in Ethiopia.

**Comment 2:** -Lines 74-75, the study objective is unclear. What do you mean by the level?

**Response 2:** I have edited the objective of the study in the manuscript. Level in this study refers to maternal healthcare services during pregnancy, during delivery and during postnatal.

**Materials and methods**

**Comment 3:** Please describe the study population more thoroughly. How many participants were reported in the DHS data, and how many were included in your study?

**Response 3:** I have described the study population in the manuscript.

**Comment 4**: The authors should describe how they reached the final sample size for their analysis.

**Response 4:** I have edited it in the manuscript. I have not applied any sample size calculation here, but I took all population in the dataset after generating appropriate outcome variables based on their operational definition. Sample size throughout the continuum decreases because the subsequent outcome builds on the success of previous level.

**Comment 5:** Please provide more details about stratified two-stage cluster sampling design of Ethiopian DHS 2016 and provide a reference for the study design. Provide more details about each stage, such as what are the sampling units, how many units are selected and out of how many, how are those units selected.

**Response 5: The comment accepted and the detail included** in the manuscript

**Comment 6:** Outcome variables: What is the basis of defining three components of CoC? Provide the references or the basis for the definition.

**Response 6:** The basis for the definition three components in CoC is WHO recommendation and other reference. Antenatal care, skilled birth attendants, and postnatal care are regarded as crucial maternal health-care (MHC) services for improving many health outcomes of mothers and babies. They are vital interventions and effective to save maternal and child life; reduce morbidity and mortality**.**

**Comment 7:** Statistical analysis: It is not clear what you are referring to two-level multilevel logistic analysis. What are the different levels? The DHS has a complex survey design; however, your data analysis seems to ignore the survey design. Please provide more details about the statistical analysis.

**Response 7:** For the purpose of clear I added some information the manuscript about statistical analysis. I have used two-level multilevel binary logistic regression. The two levels refer to individuals women (level-1) were nested within communities (level-2). The objective is to check whether MHC service varies because of e.g. begin in different regional state of the country.

Concerning complex survey design, I have considered the survey design during my analysis using STATA. The **melogit** command used with [iw=weight] ||psu: where psu indicates primary sampling unit.

**Discussion**

**Comment 8**: In general, this section does not clearly present the authors’ conclusions and make some broad generalizations that are not based on the data presented. The author should discuss their findings, mainly why the factors associated with CoC are particularly important in Ethiopia.

Response 8: I hope my discussion based on the study finding and compared with other previous study finding then justification was given on each paragraph. I accept the comment on some broad discussion and modified in the manuscript.

**Comment 9:** The authors should elaborate the study limitations.

**Response 9:** the limitation elaborated in the manuscript.

**Reviewer #3:**

**Comment 1:** A thorough English Editing is required.

**Response 1:** The manuscript edited for English language.

**Comment 2:** From Table 1 to 3 Sex of Household should b as Household dead

**Response 2:** It is edited as Sex household head

**Comment 3:** All tables Women Employee status should be correct as Women's Employment Status; most tables need to define p values in notes.

**Response 3**: I have done it in the manuscript.

**Comment 4**: Authors have not used various interactions of independent variables e.g. mother education interact with wealth index.

**Response 4:** I have checked multi-collinearity for all independent variables using VIF in the STATA and I could not find any.

Comment 5: I think multinomial logistic regression model is more appropriate to see ANC and then TBA effect.

**Response 5:** Thank you for your suggestion, there is the possible to apply multinomial logistic regression if the outcome variable is MHC with categorized as ANC, SBA and PNC. But my research question is completely different from this condition. So I cannot apply multinomial logistic regression here.

**Comment 6**: It is not clear Whether skilled birth attendance (SBA) include Institutional Delivery i.e. took place at a medical Centre/hospital. If a very large proportion of births are institutional then SBA or TBA term is very misleading.

**Response 6:** SBA does not include delivery attended by TBA. In Ethiopia we do have SBA at home. That means all SBA expected to occur at health institution

**Comment 7:** Discussion needs a great deal of work.

**Response 7:** I have made modification of discussion in the manuscript.
**Comment 8:** Also they need to elaborate what is the difference between continuation and continuum care.

**Response 8:** Continuum of care is the conceptual framework that recommended by WHO to monitor continuation of maternal neonatal and child healthcare service at each stage( pregnancy, delivery, postnatal and immunization).

**Reviewer #4**:

**Comment1:** Can be published without modifications. The paper investigates the level of continuity of maternal care and identify the 30 factors affecting the CoC in Ethiopia. It is a well written and well analyzed paper.

**Response 1:** Thank you very much.
